# Vitamin D Supplementation and Clinical Outcomes in Severe COVID-19 Patients—Randomized Controlled Trial

**DOI:** 10.3390/nu15051234

**Published:** 2023-02-28

**Authors:** Josipa Domazet Bugarin, Svjetlana Dosenovic, Darko Ilic, Nikola Delic, Ivana Saric, Ivo Ugrina, Sanda Stojanovic Stipic, Bozidar Duplancic, Lenko Saric

**Affiliations:** 1 Department of Anesthesiology, Reanimatology and Intensive Care, University Hospital Split, 21000 Split, Croatia; 2 Intellomics Ltd., 21000 Split, Croatia

**Keywords:** vitamin D, ICU, COVID-19, respiratory support, mechanical ventilation, ARDS

## Abstract

COVID-19 symptoms vary from asymptomatic cases to moderate and severe illness with patients needing hospitalization and intensive care treatment. Vitamin D is associated with severity of viral infections and has an immune-modulatory effect in immune response. Observational studies showed a negative association of low vitamin D levels and COVID-19 severity and mortality outcomes. In this study, we aimed to determine whether daily supplementation of vitamin D during intensive care unit (ICU) stay in COVID-19 patients with severe illness affects clinically relevant outcomes. Patients with COVID-19 disease in need of respiratory support admitted to the ICU were eligible for inclusion. Patients with low vitamin D levels were randomized into one of two groups: the intervention group received daily supplementation of vitamin D and the control group did not receive vitamin D supplementation. In total, 155 patients were randomized: 78 into the intervention group and 77 into the control group. There was no statistically significant difference in number of days spent on respiratory support, although the trial was underpowered for the main outcome. There was no difference in any of the secondary outcomes analyzed between two groups. Our study suggests no benefit in vitamin D supplementation to patients with severe COVID-19 disease admitted to the ICU and in need of respiratory support in any of the analyzed outcomes.

## 1. Introduction

Since December 2019, millions of people have been infected with novel coronavirus (SARS-CoV-2). According to the World Health Organization (WHO), there is more than 250 million confirmed cases of coronavirus disease 2019 (COVID-19) with more than 5 million confirmed deaths across the world [1].

The COVID-19 symptoms vary from asymptomatic cases and mild forms of disease which do not require special medical care to moderate and severe illness with patients needing hospitalization [1,2]. A number of patients requiring hospitalization will need some form of respiratory support and intensive care treatment (ICU) [1,2]. There are many risk factors associated with disease progression. Elderly and frail patients are at the most risk for adverse outcomes and complications. Furthermore, risk for developing complications of COVID-19 disease is increased in patients with cardiovascular disease, diabetes, malignant disease or obesity [3,4,5]. One of the factors associated with worse outcomes, severity and more complications from respiratory infections is vitamin D deficiency [6,7]. Vitamin D is known to have immuno-modulatory effects in innate and adaptive immune response [6]. It induces the production of antimicrobial proteins and could act as an anti-inflammatory agent [8,9]. It decreases viral replication rates and reduces the synthesis of proinflammatory cytokines [8,9]. Vitamin D is also known to regulate various thrombotic pathways [10]. Vitamin D supplementation could, therefore, have a role in reducing the coagulopathy associated with COVID-19 [10]. Low levels of vitamin D are associated with increased incidence of respiratory infections [11]. Studies have shown that patients with vitamin D levels <50 nmol/L had a 64% higher risk of community-acquired pneumonia [12]. Low vitamin D levels were associated with increased rates of infections, sepsis and mortality [13,14,15]. Observational studies showed the association of low levels of vitamin D with COVID-19 susceptibility, severity and mortality outcomes [13,14]. The severity of hypovitaminosis D was also found to be associated with the prognosis of COVID-19, since patients with lower levels of vitamin D were more prone to severe illness and had higher mortality rates [2,5,16,17].

There is evidence that vitamin D supplementation protects against respiratory tract infections [18]. However, there is conflicting evidence about the beneficial effects of vitamin D supplementation in COVID-19 patients. While some studies found a reduction in disease severity and earlier recovery with vitamin D supplementation, others showed no difference in outcomes [13,19,20,21,22,23,24,25].

To improve vitamin D status, high dose supplementation is required [21,26]. There are a variety of doses and dosing intervals used for vitamin D supplementation. Bolus doses with longer intervals are not recommended due to the higher incidence of adverse effects [27]. Instead, daily supplementation has been shown reduce the incidence of respiratory tract infections in the general population [28]. Regarding patients in the ICU, usual doses are insufficient, and it takes too long time to increase vitamin D levels [29]. Therefore, a loading dose or higher daily doses are necessary to correct vitamin D hypovitaminosis in timely manner [29,30]. It is known that different dosing regimens could have different clinical effects [29,30]. Daily doses lead to stable and consistent availability of vitamin D and its metabolites, which could have an impact on clinical outcomes [29]. There is currently no consensus on the upper level of vitamin D supplementation. Usual daily doses range from 400 to 2000 IU daily [31]. Endocrine society recommends an upper daily limit of 10,000 IU [32]. Studies have shown that daily supplementation of vitamin D in doses of up to 10,000 IU over longer periods of time did not cause adverse effects in humans [33,34]. Furthermore, rare side effects and a wide safety margin make vitamin D supplementation an inexpensive and safe intervention for hospitalized patients [33,34,35]

In this study, we aimed to determine whether daily supplementation of vitamin D during intensive care unit (ICU) stay in COVID-19 patients with severe illness affects clinically relevant outcomes.

## 2. Materials and Methods

This was a single center, open label randomized clinical trial. The study took place in a tertiary hospital center from November 2021 to May 2022.

### 2.1. Patients

All patients admitted for the first time to the ICU in the study period were tested with a polymerase chain reaction (PCR) test for SARS-CoV-2 infection upon admission. Patients who had positive PCR test were considered eligible for inclusion in the study if they had a need for invasive or non-invasive respiratory support. All these patients had vitamin D levels measured on admission. Patients with low levels of vitamin D (<50 nmol/L) were included in further study. All patients were older than 18 years and had confirmed COVID-19 disease with PCR test.

Non-inclusion criteria included patients with known vitamin D supplementation prior to hospitalization, patients with chronic hepatic dysfunction or intestinal malabsorption syndromes, concomitant use of medication affecting vitamin D metabolism or absorption (anticonvulsants, etc.), elevated serum calcium levels on admission (corrected for albumin concentration), history of hypercalciuria and kidney stones, conditions that could lead to high serum calcium levels (sarcoidosis, tuberculosis, lymphomas), pregnancy or breastfeeding, chronic dialysis, severe chronic kidney disease (estimated glomerular filtration rate (eGFR) < 30 mL/min/1.73 m^2^) and parathyroid disease.

Patients were excluded from study in case of calcium levels that were consistently above normal serum range (>2.6 mmol/L, >10.5 mg/dL) or if vitamin D levels were >150 nmol/L.

### 2.2. Intervention

All patients included in this study received standard of care. Mechanical ventilation was applied with protective lung ventilation using tidal volumes 4–8 mL/kg and plato pressures ≤ 30 cm H_2_O. Dexamethasone was routinely administered to all eligible patients. In cases of moderate and severe acute respiratory distress syndrome (ARDS) when adequate oxygenation could not be achieved, patients were ventilated in prone position in the early phase of ICU stay according to the Berlin definition [36,37]. Contraindications for prone positioning were recent cardiac, abdominal, thoracic or tracheal surgery, burns, pregnancy, unstable fractures and spinal instability. Patients randomized into the intervention group received 10,000 IU of cholecalciferol daily (2.5 mL of Plivit D_3_ © 4000 IU oral suspension 4000 IU/mL, Pliva). Supplement was administered orally or via gastric tube during ICU stay or for at least 14 days in case of ICU discharge before day 14. Supplementation was started within 48 h of admission to ICU. Supplement was administered by experienced nursing staff. For patients receiving supplementation, vitamin D levels were checked on days 7 and 14. In case that vitamin D levels were >150 nmol/L or if the calcium levels were consistently >2.6 mmol/L, further supplementation was stopped.

### 2.3. Randomization

Patients were randomly assigned into one of the two groups. The intervention group received vitamin D supplementation, and the control group did not receive vitamin D supplementation. Patients from both groups received standard of care. Patients were randomized in a 1:1 ratio to the intervention or control group. A randomization list was created using a computer-generated code with block sizes of 10 (https://www.graphpad.com/, accessed on 30 September 2021) by one of the study investigators.

### 2.4. Blinding

The person assigned for data analysis was blinded as to which group received intervention.

### 2.5. Outcomes

Primary outcome was number of days spent on respiratory support (invasive or non-invasive).

Secondary outcomes were all-cause mortality on days 14, 28 and 60, clinical improvement at day 28 (WHO clinical progression scale), number of days spent in ICU, number of days spent in hospital, bacterial superinfections, neutrophil to lymphocyte ratio and disease severity (CRP levels, PaO_2_/FiO_2_ ratio, D-dimer levels, fibrinogen, ferritin, PCT). All patients were monitored for adverse effects of vitamin D supplementation.

### 2.6. Assessments (Primary, Secondary Outcomes)

Data were obtained from patient electronic and paper medical records by a critical care physician. Data collection included demographic data, comorbidities, vaccination status, chronic therapy, vitamin D levels on admission, disease severity markers (arterial partial pressure of oxygen/fraction of inspired oxygen ratio (PaO_2_/FiO_2_), C reactive protein (CRP), D-dimer, fibrinogen, ferritin, procalcitonin (PCT) levels, neutrophil to lymphocyte ratio, and severity organ failure score (SOFA)). All data were collected upon admission and before randomization. Randomization was performed within 48 h of admission.

25-OH vitamin D serum levels are not routinely measured in all ICU patients. For the purpose of this study, vitamin D levels were measured in a hospital laboratory using the ECLIA method (Elecsys Vitamin D total III kit; Roche Diagnostics, Diegem, Belgium). Vitamin D levels for patients included in this study were measured on admission to the ICU, and for patients randomized into the intervention group, vitamin D levels were checked on days 7 and 14 of ICU or hospital stay. For patients in the control group, no further vitamin D levels were measured.

Disease severity markers were collected daily during ICU stay and every third day after discharge from ICU. Assessment for bacterial superinfections included taking blood, urine and tracheal aspirations for microbiology testing. Samples were taken upon admission, on day 5, and further based on clinical and laboratory signs of possible bacterial infection. In case of patients discharge from hospital before the 28-day period, patients were contacted by phone on day 28 and day 60.

### 2.7. Sample Size

Since the primary outcome was number of days spent on respiratory support, a difference of two days was set arbitrarily as a difference that would mean significant clinical effect. Considering that, the calculated sample size to detect a 2-day difference in number of days on respiratory support was 274 (137 patients in each group). However, the number of severe COVID-19 patients treated in the ICU in the study period was large; almost half of them did not meet inclusion criteria. Furthermore, the number of patients admitted to the ICU declined during the study period, so we were not able to include the planned number of patients.

### 2.8. Statistical Analysis

Data analysis was performed using the R Software (version 4.2.1) [38]. Usual descriptive statistics was performed with means, medians, standard deviations, boxplots, etc. Categorical variables were expressed as numbers, percentages or factors in modeling in R. Continuous variables were presented as means, medians and standard deviations in tables and presented through box plots on graphs. A two-sample test of proportions was used to determine whether the two populations differ significantly in survival on a specific day. A Mann–Whitney–Wilcoxon test was used to test the difference between laboratory parameters. FDR correction for *p*-values was applied, and *p*-values with *p* < 0.05 were taken as significant. Additionally, LOESS was utilized for graphs [39].

### 2.9. Consent Procedure

Since all patients admitted to our ICU were sedated and/or intubated and it was not possible to obtain informed consent from them, a consent for participation in this study was obtained from family members over the phone before patients’ inclusion in the study.

### 2.10. Ethical Considerations

This study was conducted in accordance with the Declaration of Helsinki of the World Medical Association for experiments involving humans. The study was approved by the institutional ethical board (Ethical Committee of University Hospital Split, Croatia; approval number: 500-03/21-01/170).

The study was registered in ClinicalTrials.gov (NCT05384574).

### 2.11. Reporting

This study is reported in accordance with the CONSORT Statement [40].

## 3. Results

There were 292 patients with COVID-19 disease admitted to the ICU from November 2021 to May 2022. Of those, 137 patients had one or more non-inclusion criteria met, leaving 155 patients eligible for randomization. Of those, 78 patients were randomized into the intervention group and 77 patients were randomized into the control group. Three patients from the intervention group were excluded because they did not receive vitamin D supplementation according to the study protocol. A study flow diagram is presented in Appendix A. Demographic data of randomized patients are presented in Table 1. All patients included in this study were contacted by phone two to three months after hospital discharge.

Regarding the primary outcome results, the median number of days on respiratory support was 9 (IQR 6–16). The median number of days on respiratory support for the intervention group was 10 (IQR 6–13.9) and for the control group was 8 (IQR 6–12). There was no statistically significant difference in primary outcome between the two groups.

### Secondary Outcomes

Overall 14-, 28- and 60-day survival rates were 87.8% (130/148 patients, data for four patients were unavailable), 71.3% (102/143 patients, data for nine patients were unavailable) and 66.4% (83/125 patients, data for 14 patients were unavailable), respectively. There was no statistically significant difference in neither of these survival rates between the two groups.

The median number of days spent in the ICU for both groups was 13 (IQR 9–20) and the median number of days in the hospital was 19 (IQR 14–26). The median PaO_2_/FiO_2_ ratio on admission was 75 (IQR 64–93). The mean WHO clinical progression scale on admission was 8.4 (SD ± 0.5). Results for the intervention and control groups are presented in Table 2.

Regarding vitamin D supplementation, the mean vitamin D level on admission was 27.1 ± 12.4 nmol/L. The mean vitamin D level for the intervention group on admission was 27.23 ± 11.8 nmol/L. There was no difference between the two groups (*p* 0.95). The mean vitamin D levels on days 7 and 14 in the intervention group were 38.5 ± 15.5 nmol/L and 56.2 ± 23 nmol/L, respectively, showing that vitamin D supplementation did increase vitamin D levels during the supplementation period. There was no difference between male and female subjects regarding vitamin D levels on admission (M 28 ± 12, F 25 ± 11, *p* 0.17).

**Figure 1:**
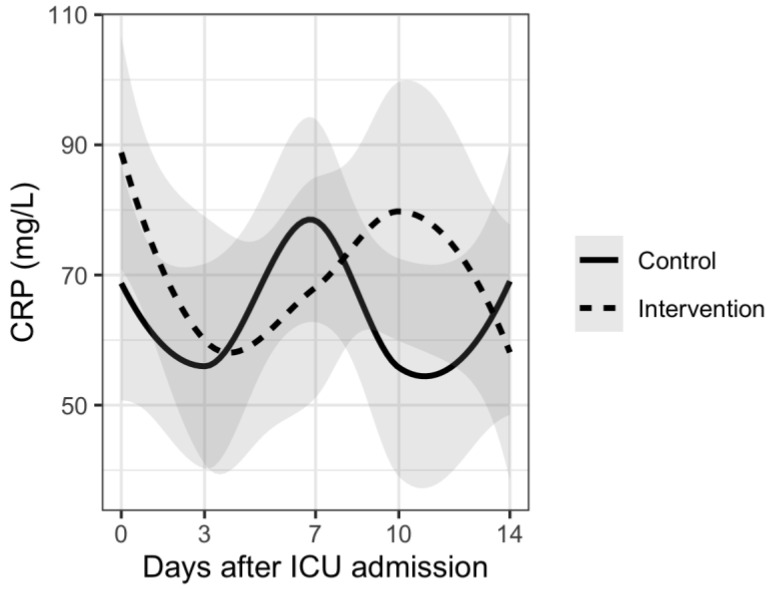
Mean CRP levels and trends during 14 days from ICU admission.

**Figure 2:**
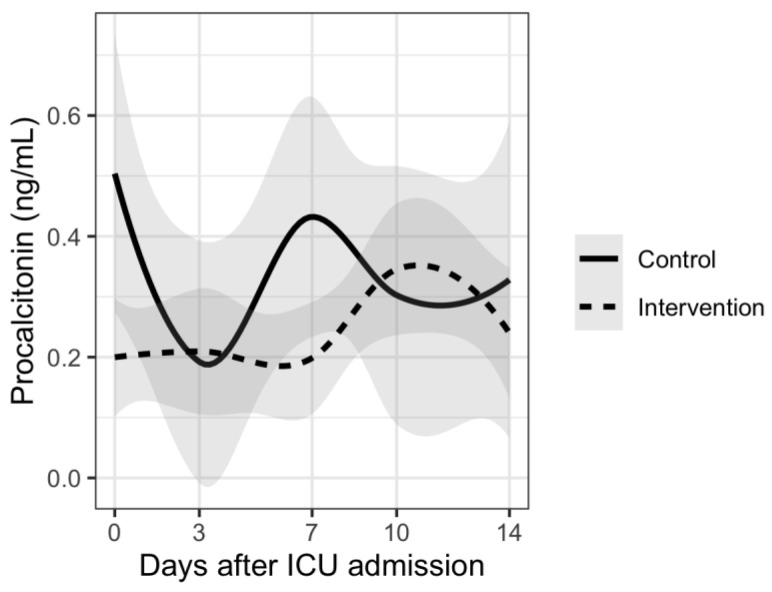
Mean procalcitonin levels and trends during 14 days from ICU admission.

**Figure 3:**
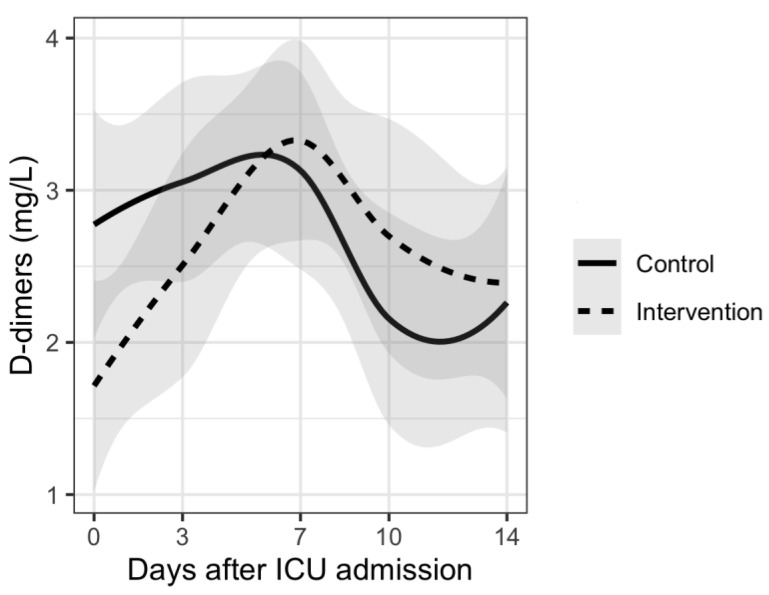
Mean D-dimer levels and trends during 14 days from ICU admission.

**Figure 4:**
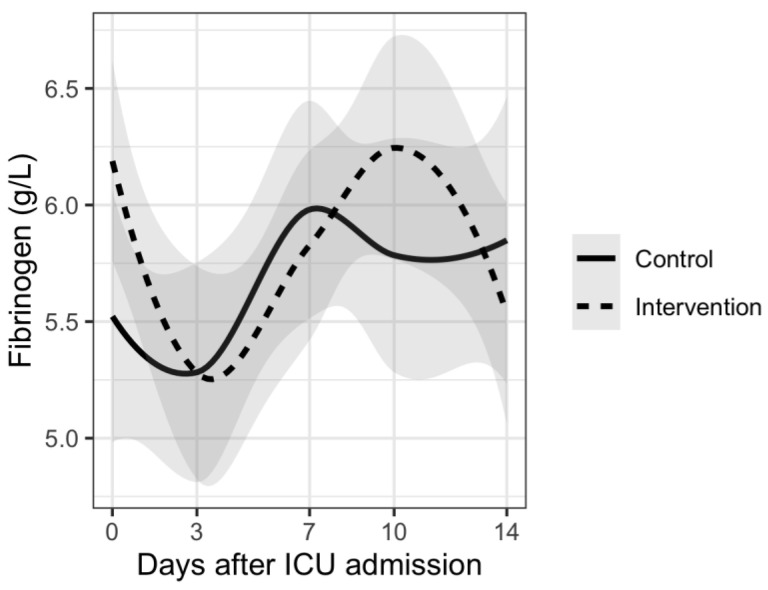
Mean fibrinogen levels and trends during 14 days from ICU admission.

**Figure 5:**
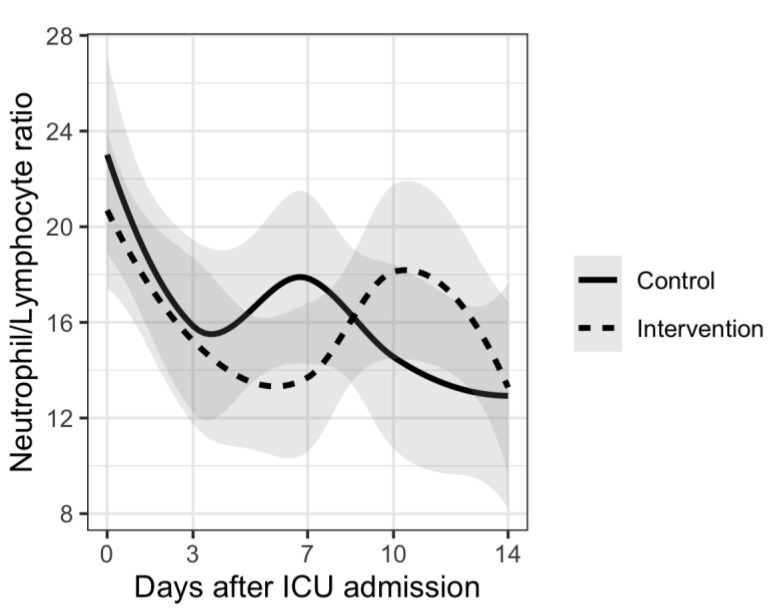
Mean neutrophil/lymphocyte ratio and trends during 14 days from ICU admission.

Analysis of disease severity markers showed differences in trends for CRP, PCT and neutrophil/lymphocyte ratio. Patients in the intervention group had delayed increases in CRP and PCT plasma levels and also in neutrophil/lymphocyte ratio compared to the control group. However, there were no significant differences between the two groups. Results are shown in Figure 1, Figure 2, Figure 3, Figure 4 and Figure 5.

No adverse effects attributable to vitamin D supplementation were observed during this study. None of the patients in the intervention group met exclusion criteria.

## 4. Discussion

In our study, vitamin D supplementation in patients with severe COVID-19 treated in an intensive care unit (ICU) for invasive or non-invasive respiratory support did not reduce the number of days on respiratory support compared to patients who did not receive vitamin D supplementation. Furthermore, there were no differences in the number of days spent in the ICU, hospital length of stay or rate of bacterial superinfections. Regarding the survival, there were no differences in 14-, 28-day or 60-day survival rates. However, the study findings should be interpreted with caution due to inadequate study power for primary outcome. With this caveat, the trial should be viewed as a contribution to a still under-researched body of evidence about the effect of vitamin D supplementation in critically ill patients with COVID-19. The results of this RCT should be pooled in a systematic review, since a single trial can seldom provide a definitive answer [41]. Vitamin D deficiency is defined by the Endocrine Society Task Force on Vitamin D as vitamin D levels <50 nmol/L [32]. The prevalence of vitamin D deficiency varies from region to region from 24% to 40% [42,43,44]. In critically ill patients, this prevalence is even higher and is associated with greater mortality and morbidity in both pediatric and adult ICU patients [45].

Vitamin D supplementation was shown to have benefits regarding acute respiratory infections [28,46]. However, dosage and dosing intervals have not been clearly defined, and there are various administration protocols [25,28,30,47,48,49,50]. High-dose enteral or parenteral vitamin D supplementation did not show a clear benefit on various clinical outcomes in a number of studies [23,47,48,49]. On the other hand, there are studies showing that daily vitamin D supplementation had a positive effect on respiratory tract infections [46]. Furthermore, a systematic review by Pal et al. showed that vitamin D supplementation improves clinical outcomes in COVID-19 patients [20]. The majority of the analyzed studies used oral cholecalciferol with different cumulative doses ranging from 80,000 to 400,000 IU [20]. This study also showed that there was no difference in outcomes between high and low cumulative doses, with cumulative doses less than 200,000 IU categorized as low dose [20].

In this study, vitamin D supplementation was administered in daily doses of 10,000 IU of cholecalciferol. This dosage increased mean values of vitamin D concentrations from 27.2 to 56.2 nmol/L over a 14-day period, and no adverse effects were recorded. Studies showed that these doses are safe, and the cumulative dose for patients included in our study is considered low [20]. This approach also could lead to a greater availability of vitamin D metabolites, which could have a greater positive effect [29].

Results for the primary outcome in our study show that vitamin D supplementation did not reduce the number of days on respiratory support (median number of days 10 (IQR 6–15) in the intervention group and 8 (IQR 6–15) in the control group). These results are in concordance with the results published by Lan et al. in their systematic review [22]. Furthermore, similar results were published by other authors in several RCTs [23,47]. In both studies, vitamin D was given in a single dose and in much higher dose than the one used in our study.

Regarding secondary outcomes, we found no significant differences in the number of days spent in the ICU nor in hospital length of stay. Most of the published studies showed similar results [5,20,23,47,48,49,51]. Lan et al. showed in their systematic review that numerical results were better in the vitamin D supplementation group [22]. However, these results did not reach statistical significance. In another SR, the authors showed a potential beneficial use of vitamin D intervention in COVID-19 in a pooled analysis of six RCTs [51]. However, no significant effect was found for individual outcomes of ICU care and mortality [51].

In this study, there was no difference in the 14-, 28- or 60-day survival rate between the intervention and control group. Bassatne et al. showed in their systematic review increased mortality rates in COVID-19 patients with vitamin D deficiency [5]. In studies analyzing whether vitamin D supplementation improves survival in COVID-19 patients, the results did not show positive effect regardless of the dosage and methods of administration [20,23,48,49].

A systematic review by D’Ecclesiis et al. included 38 studies that assessed the association of vitamin D levels or vitamin D supplementation with SARS-CoV2 infection, severity and mortality [52]. The authors showed improved outcomes, including a halved risk of COVID-19 severity (measured as need for ICU admission or ventilation or intubation) and mortality with vitamin D supplementation [52]. The authors also pointed out some important limitations, such as the results were based mainly on observational studies with potential for bias, the possibility of unmeasured confounding factors such as different type of care in hospitals, and significant heterogeneity among studies observed, concluding that RCTs are needed to establish the causal link [52].

The results of our study show different trends in disease severity markers between patients who received vitamin D supplementation and those who did not receive supplementation. Many of the patients from our study had increases in inflammatory and disease severity markers 7–10 days after ICU admission. This can be explained by the fact that more than 70% of patients from both groups had bacterial superinfection during their ICU stay. There was no significant difference between the two groups. Similar results were reported in a rapid review where authors report that supplementing vitamin D did slightly improve fibrinogen levels but did not influence D-dimer, CRP or PCT levels [48]. Although there was no statistically significant difference, patients from the intervention group had delayed increases in CRP and PCT levels as well as in neutrophil/lymphocyte ratio. These results could suggest a potential benefit of vitamin D supplementation regarding nosocomial infections in ICU. However, no conclusions can be made based on our results.

### Limitations

The main limitation of our study is the small number of patients included. We planned to include at least 274 patients (137 in each group) in order to detect a 2-day difference in the number of days on respiratory support. However, many of the patients admitted to the ICU in our study period met one of the non-inclusion criteria and were not eligible for randomization. Furthermore, since the characteristics of the pandemic changed and there were lower rates of hospital admissions and ICU admissions, we were unable to reach the calculated sample size. Therefore, the results of this trial need to be further corroborated in larger, adequately powered RCTs.

The second limitation is the single center design, which could influence the external validity of the study. Large multicenter studies would obtain more conclusive evidence.

Our patients had a median PaO_2_/FiO_2_ ratio of 75 mmHg, which means that many of the patients admitted to the ICU had severe ARDS. It may be possible that vitamin D supplementation does not have a positive effect at this critical stage of illness, which does not mean that it could not have a beneficial effect at earlier stages of the disease.

## 5. Conclusions

In this RCT, the daily supplementation of vitamin D in severe COVID-19 patients admitted to the ICU and who need respiratory support did not seem to reduce the number of days on respiratory support. Furthermore, the results suggest no statistically significant difference in any of the secondary outcomes. The results should be interpreted with caution, and further research is needed, since the trial was underpowered for the main outcome.

Results from our study are similar to results published by other authors. It seems that vitamin D supplementation does not significantly improve clinical outcomes in severe COVID-19 patients regardless of dosage and methods of administration. However, there could be a potential benefit of vitamin D supplementation in less severe COVID-19 patients. Further studies on a larger number of patients are needed to determine whether vitamin D supplementation has an effect on severe COVID-19 patients.

## Figures and Tables

**Table 1:** Demographic data of patients, vaccination status, vitamin D levels, PaO_2_/FiO_2_ ratio, WHO clinical progression scale on admission to ICU.

	Total	Intervention Group	Control Group
Number of patients	152	75	77
Gender			
Male	110 (72.4)	52 (69.3%)	58 (75%)
Female	42 (17.6)	23 (30.7%)	19 (25%)
Age, years (mean, min, max)	64.95 (39–82)	65 (59–71)	65.5 (39–82)
Vaccinated	37 (24.3)	20 (26.7%)	17 (22%)
Vitamin D levels, nmol/L	24.4 (16.95–36.8)	25.3 (17.9–36.9)	27.3 (16–37.3)
PaO_2_/FiO2, mmHg	75 (65–93)	75 (65–93)	75.5 (65.2–92.5)
WHO clinical progression scale	8 (8–9)	9 (8–9)	9 (8–9)
Comorbidities			
Hypertension	69 (45.4%)	31 (41.93%)	38 (49.3%)
Diabetes mellitus	42 (27.6%)	21 (28%)	21 (27.3%)
Cardiovascular disease	34 (22.4%)	14 (18.7%)	20 (26%)
Cerebrovascular disease	8 (5.3%)	5 (6.7%)	3 (4%)
Chronic lung disease	15 (9.9%)	8 (10.7%)	7 (9.1%)
Malignant disease	9 (5.9%)	7 (9.3%)	2 (2.6%)
Autoimmune disease	37 (24.3%)	19 (25.3%)	18 (23.4%)
Number of comorbidities			
No comorbidities	28 (18.4%)	15 (20%)	13 (16.9%)
1	58 (38.2%)	29 (38.7%)	29 (37.7%)
2	44 (28.9%)	20 (26.7%)	24 (31.1%)
≥3	22 (14.5%)	11 (14.7%)	11 (14.3%)

Data are presented as numbers (percentages) or median (interquartile range); PaO_2_/FiO_2_, ratio of arterial oxygen partial pressure to fractional inspired oxygen; WHO, World health organization.

**Table 2:** Outcomes of intervention and control group.

	Intervention Group	Control Group	*p* Value
Number of days on respirator	10 (6–15)	8 (6–15)	0.283
Number of days in ICU	13 (9–20)	12 (8–20)	0.515
Number of days in hospital	19 (14–25.8)	18 (12–25)	0.757
WHO clinical progression scale on admission	8 (8–9)	8 (8–9)	0.736
WHO clinical progression scale on day 28	5 (0–10)	5 (0–10)	0.705
Survival at day 14 (%)	66 (89)	64 (86.5)	0.606
Survival at day 28 (%)	52 (75.4)	50 (71.4)	0.561
Survival at day 60 (%)	45 (73.8)	38 (59.4)	0.131
Number of bacterial superinfections (n %)	56 (74.6)	55 (71.4)	0.789

Data are presented as median (interquartile range) and numbers (percentage); ICU, intensive care unit; WHO, World Health Organization.

## Data Availability

Data is available on request.

## Appendix A

Study flow diagram.
